# A Novel *SCN9A* Mutation Responsible for Primary Erythromelalgia and Is Resistant to the Treatment of Sodium Channel Blockers

**DOI:** 10.1371/journal.pone.0055212

**Published:** 2013-01-31

**Authors:** Min-Tzu Wu, Po-Yuan Huang, Chen-Tung Yen, Chih-Cheng Chen, Ming-Jen Lee

**Affiliations:** 1 Graduate Institute of Zoology, College of Life Sciences, National Taiwan University, Taipei, Taiwan; 2 Institute of Biomedical Sciences, Academia Sinica, Taipei, Taiwan; 3 Department of Neurology, National Taiwan University Hospital, Tapei, Taiwan; 4 Medical Genetics, National Taiwan University Hospital, Tapei, Taiwan; Virginia Commonwealth University, United States of America

## Abstract

Primary erythromelalgia (PE) is an autosomal dominant neurological disorder characterized by severe burning pain and erythema in the extremities upon heat stimuli or exercise. Mutations in human *SCN9A* gene, encoding the α–subunit of the voltage-gated sodium channel, Na_v_1.7, were found to be responsible for PE. Three missense mutations of *SCN9A* gene have recently been identified in Taiwanese patients including a familial (I136V) and two sporadic mutations (I848T, V1316A). V1316A is a novel mutation and has not been characterized yet. Topologically, I136V is located in DI/S1 segment and both I848T and V1316A are located in S4-S5 linker region of DII and DIII domains, respectively. To characterize the elelctrophysiological manifestations, the channel conductance with whole-cell patch clamp was recorded on the over-expressed Chinese hamster overy cells. As compared with wild type, the mutant channels showed a significant hyperpolarizing shift in voltage dependent activation and a depolarizing shift in steady-state fast inactivation. The recovery time from channel inactivation is faster in the mutant than in the wild type channels. Since warmth can trigger and exacerbate symptoms, we then examine the influence of tempearture on the sodium channel conduction. At 35°C, I136V and V1316A mutant channels exhibit a further hyperpolarizing shift at activation as compared with wild type channel, even though wild type channel also produced a significant hyperpolarizing shift compared to that of 25°C. High temperature caused a significant depolarizing shift in steady-state fast inactivation in all three mutant channels. These findings may confer to the hyperexcitability of sensory neurons, especially at high temperature. In order to identifying an effective treatment, we tested the IC_50_ values of selective sodium channel blockers, lidocaine and mexiletine. The IC_50_ for mexiletine is lower for I848T mutant channel as compared to that of the wild type and other two mutants which is comparable to the clinical observations.

## Introduction

Erythromelalgia (or erythermalgia; OMIM 133020) is a rare neurovascular pain disorder characterized by intermittent severe burning pain, erythema and elevation of temperature in the extremities. It was first named and described in 1878 by Dr. Mitchell [1]. The symptoms are usually bilateral and symmetrical, and they are most often confined in lower extremities but can extend to hands and sometimes earlobes and nose tip [2]. Primary erythromelalgia (PE, or inherited erythromelalgia, IEM) can be hereditary or sporadic. Familial PE transmitted by an autosomal dominant manner. The age of onset for PE is usually before the first decade of life (as early as months after birth) but can also be adult onset [3]. The symptoms appear to persist and progressively worsen throughout life for most patients, although some patients reported to show improvement even complete resolution of symptoms [4]. The painful attacks can be evoked by warm stimuli and moderate exercises. It was described that the critical temperature lies within the range of 32–36°C [5]. Patients therefore are compelled not to wear socks or closed shoes even in winter. An attack can last from minutes to hours and even days in some cases. Patients can relieve symptoms by elevating affected limbs or submerging affected areas in cold water but by doing so too often, patients might suffer from skin infections. There has not been an effective treatment for PE. In a report of 168 patients with erythromelalgia, 84 medications or treatments (sodium channel blockers, sympathetic blocks, etc.) were used and showed high variability and no treatment is consistently effective [4].

Genetic linkage locus was found at chromosome 2 from a large kindred of five generations with multiple family members affected with PE [2]. Analysis of recombination events identified the D2S2370 and D2S1776 as flanking markers in chromosome 2q31-32 [2]. Subsequently Yang *et* *al.* further refined the linkage to a 5.98 cM region in chromosome 2q24.2-q24.3. By sequencing the selected candidate genes, two missense mutations (I848T, L858F) were found in *SCN9A* gene in the family and a sporadic patient with PE [6]. Human *SCN9A* gene contains 26 exons and encodes for the α-subunit of voltage-gated sodium channel, Na_v_1.7. To date 19 mutations in *SCN9A* gene have been reported relating to PE, including 18 missense mutations causing single amino acid substitution and one in-frame deletion. The penetrance is nearly 100% for PE mutations.

**Figure 1:**
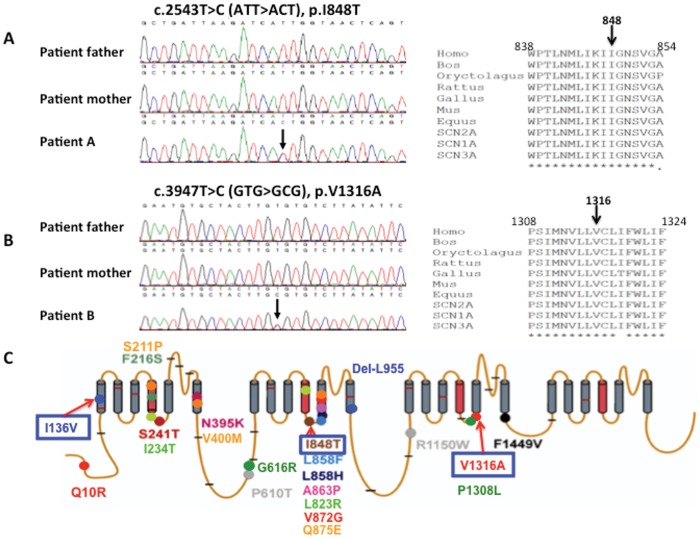
Sequence chromatography of patients with I848T and V1316A mutations in *SCN9A* gene, and molecular topology with the location of mutations of human Na_v_1.7. (A) A point mutation c.2543T>C in exon 15 results in a missense mutation of isoleucine 848 changed to threonine was found in patient A, but not her parents. Amino acid sequence alignment for I848T in the right panel demonstrates this amino acid is highly conserved among different species and variable sodium channels. (B) A point missense mutation of c.3947T>C in exon 22 results in valine 1316 changed to alanine in patient B. Her parents do not harbor the sequence variant. The sequence alignment demonstrates the highly conserved amino acid, isoleucine1316. (C) There are 19 missense and one in-frame deletion mutations that have been identified so far relating to primary erythromelalgia. The gray annotations are polymorphisms that are not directly linked with PE. Mutations analyzed in this study are denoted with blue boxes. (This figure is modified from a figure previously published by Waxman and Dig-Hajj (2005) in “Erythermalgia: molecular basis for an inherited pain syndrome”, Trends in Molecular Medicine, 11(12), 555–562 [37].).

Voltage-gated sodium channel (Na_v_) comprises of a family of nine structurally related pore forming α-subunits (Na_v_1.1–1.9) and a family of accessory β-subunits (β1– β4). These channels are encoded by *SCN1A – SCN5A* and *SCN8A – SCN11A* genes for the α-subunits, and *SCN1B – SCN4B* genes for the β-subunits. The α-subunit is a ∼260 kDa pore forming protein consisting of four homologous domains (DI – DIV) and six transmembrane segments (S1– S6) in each domain (Fig. 1).

Na_v_1.7 is composed of 1977 amino acids (∼225 kDa), which is predominantly expressed in dorsal root ganglion (DRG) neurons and sympathetic ganglion neurons [7]. It produces rapid activating and inactivating current that is sensitive to TTX blockade [8]. In the DRG, Na_v_1.7 is concentrated in small C fiber nociceptors and to a lesser extent in medium-sized Aδ and large Aβ cells [9]. Immunohistochemical studies showed that Na_v_1.7 is present at the distal ends of neurites, close to the impulse trigger zone where neuronal firing is initiated [10]. Na_v_1.7 is characterized by slow recovery from inactivation and slow closed-state inactivation, which allows Na_v_1.7 channels to respond to slow ramp depolarizations and amplify generation potentials at nerve terminals [10], [11]. Na_v_1.7 is therefore like a “threshold channel” that amplifies small, subtle depolarizations and brings membrane potential to more depolarized state to activate Na_v_1.8, which is responsible for most of the rising phase of action potentials. Na_v_1.7 is poised as a molecular gatekeeper of pain detection at peripheral nociceptors [10].

In addition to PE, mutations in *SCN9A* gene can also result in paroxysmal extreme pain disorder (PEPD; OMIM 167400) [12] and congenital insensitivity (or indifference) to pain (CIP; OMIM 243000) [13]. PEPD is an autosomal dominant pain disorder caused by gain-of-function mutations in *SCN9A* gene, but CIP resulted from loss-of-function and transmitted in a recessive pattern. Furthermore, gain-of-function mutations in *SCN9A* gene were found to be associated with idiopathic small fiber neuropathy (I-SFN) [14]. Eight out of 28 patients (28.6%) with I-SFN were screened positive for novel mutations in *SCN9A* gene and subsequent electrophysiological analyses revealed multiple gain-of-function changes in the mutant channels. Each of the mutations confers to the hyperexcitable DRG neurons. The results suggest an etiological basis for I-SFN, whereby expression of gain-of-function mutant Na_v_1.7 channels in small diameter peripheral axons, may cause these fibers to degenerate [14].

In this study we report a sporadic mutation, I848T as well as a novel mutation, V1316A in *SCN9A* gene from Taiwanese patients. Together with the previous reported I136V mutant channel, we characterized the basic electrophysiological properties and evaluated how increased temperature can modulate the gating properties of these mutant channels in CHO-K1 cells. In addition, since treatments for PE have only been partially successful and seem to be mutation specific, we assessed the differential inhibition of sodium channel blockers on these mutant channels and using the electrophysiological parameters as a tool to screen for the potential treatments in these patients.

## Materials and Methods

### Study Patients

Three mutations have recently been identified in Taiwanese patients who were diagnosed with primary erythromelalgia (PE). The diagnostic criteria for erythromelalgia include: 1) burning pain of extremities, 2) pain aggravated by warming, 3) pain relieved by cooling, 4) erythema of the affected skin, and 5) increased temperature of the affected skin [15], [16]. Differential diagnosis including reflex sympathetic dystrophy, Fabry’s disease, and other painful neuropathies associated with diabetes mellitus, alcoholism, HIV infection and Lyme disease were excluded. Written informed consent was obtained from all participants following ethical practices approved by the institutional review board of National Taiwan University Hospital Ethics Committee. The written inform consent has been obtained from the parents of the juvenile patients under 18 years of age.

### Patient A

Patient A is a 33-year-old female diagnosed with PE about three years ago at National Taiwan University Hospital, Taipei, Taiwan. She suffered from burning pain and erythema in her feet since she was eight years old. At hot weather, the feet developed severe burning pain with reddish skin discoloration. Her palms were affected as well in recent decade. Walking and warmth trigger the symptoms, which made difficulties for her to wear shoes and socks. To relieve foot pain, she preferred to have her feet immersed in water and even on ice to gain pain relief. Mexiletine and acetaminophen have been prescribed with mild improvement in pain scale. There is no family history and her parents are unremarkable.

### Patient B

Patient B was a 16-year-old girl upon her first visit to National Taiwan University Hospital. The chief complaint was the recurrent severe burning pain of both feet since age seven. The symptoms affecting both feet symmetrically, and gradually extended to bilateral knees and hands in recent months. Various medications such as aspirin, mexiletine, carbamazepine, propranolol, gabapentin, and imipramine have been prescribed but only little effect was observed. To relieve pain, she had to immerse her hands and feet in ice water at night. Nevertheless, prolonged immersion in ice-cold water with small wound resulted in infection associated with blisters and cellulitis. Local heating due to inflammation aggravated the causalgia and burning pain of both feet. Unfortunately, neurogenic shock with collapse took place due to severe pain after debridement surgery of her feet.

### Genetic Analysis

DNA was extracted from patients’ blood using Genomic DNA Extraction Kit (Geneaid, Taipei, Taiwan). Informed consent was signed before blood samples were collected. Polymerase chain reaction (PCR) was performed in thermal cycler (GeneAmp® PCR System 2700, Applied Biosystems, CA, USA) for each of the 26 coding exons. Exon primers were designed according to the published sequences (GeneBank NM_002977) as described by Yang *et* *al*. [6] The PCR reaction mixture (50 µl) contains 2 mM dNTP (5 µl), 10X PCR Buffer (5 µl), forward primer 10 µM (3 µl), reverse primer 10 µM (3 µl), 1.25U Taq DNA polymerase (Thermo, MA, USA), dH_2_O 27 µL, and DNA template 100 ng. The PCR protocol included an initial cycle of denaturation at 95°C for 15 minutes, followed by 25 cycles of (94°C for 30 seconds, 60°C for 30 seconds decreased by 0.4°C/cycle and 72°C for 45 seconds), 12 cycles of (94°C for 30 seconds, 50°C for 30 seconds and 72°C for 45 seconds) and an extension step at 72°C for 7 minutes. The PCR products were purified with PCR DNA Fragments Extraction Kit (Geneaid, Taipei, Taiwan), and sequenced using BigDye®sequencing chemistry (version III, Applied Biosystems, CA, USA) and analyzed on an automated ABI3100™ DNA sequencer (Applied Biosystems, CA, USA).

### Expression Constructs

The full-length cDNA of human *SCN9A* gene was purchased from OriGene company (OriGene Technologies, Rockville,MD, USA). It was first cloned into pTracer-EF/V5-His A vector (Invitrogen, Life Technologies, Carlsbad, CA, USA) using NotI cloning site (suppliment Fig. S1A & S1B). The GFP sequence that is driven by a separate promoter in pTracer vector allows visual identification of transfected cells when performing electrophysiological recordings. Because of the large size of *SCN9A* cDNA (∼6.4 kb), short segments containing regions of point mutations were subcloned into pBluescript vector for mutagenesis (Fig. S1C), which was done using Site-Directed Mutagenesis Kit (Stratagene, Agilent Technologies, Santa Clara, CA, USA). Each mutation-containing segment was cloned back to full-length *SCN9A* cDNA. To amplify, pTracer-SCN9A (both wild type and mutant) plasmids were transformed into JM109 competent cells and cultured at 30°C in standard ampicillin (100 µg/ml) containing LB media. In addition to the α–subunit, the β-subunit has also been constructed as pIRES-hSCN1B/2B (Fig. S2).

### Cell Line Transfection

CHO-K1 (Chinese Hamster Ovary) cells (Food Industry Research and Development Institute, Hsinchu, Taiwan) were cultured under the standard condition (5% CO_2_; 37°C) in F12 medium (Gibco®, Life Technologies) supplemented with 10% fetal bovine serum (Gibco®, Life Technologies). The wild type and mutant SCN9A constructs (wild type or mutant) were co-transfected with the pIRES-SCN1B/2B β-subunit construct (Fig. S2), in 1∶3 ratio using Lipofectamine™ 2000 (Invitrogen, Life Technologies). Electrophysiology recordings were performed within 24–48 hours post-transfection, by which the transfected cells were visualized with GFP expression under fluorescent microscope (Fig. S3).

### Whole-cell Patch-clamp Recordings

Whole-cell patch-clamp recordings were conducted at room temperature (23–25°C) as previously described [17]. (Please replace the reference with Lin et al., 2012 in PNAS) Glass pipettes (Warner Products 64-0792, Hamden, CT, USA) were prepared (7–9 MΩ) with use of a vertical puller (NARISHIGE PP-830, Tritech Research, Inc., Los Angeles, CA, USA). The Axopatch MultiClamp 700B (Axon Instruments, Molecular Devices, CA, USA) was employed for whole-cell recording experiments. The stimuli and the digital records captured would be performed with use of the Signal 3.0 software and a CED1401 converter (Cambridge Electronic Design, Cambridge, England). Currents were sampled at a rate of 10 kHz and filtered at 3 kHz. The pipette solution contained (in mM): 10 NaCl, 110 CsCl, 20 TEA, 2.5 MgCl_2_, 5 EGTA, 3 ATP, 5 HEPES, pH 7.0 (adjusted with CsOH), and the osmolarity was adjusted to 300 (±10) mOs-mol/L with glucose. The extracellular bath solution contained (in mM): 100 NaCl, 5 CsCl, 30 TEA, 1.8 CaCl_2_, 1 MgCl_2_, 0.1 CdCl_2_, 5 HEPES, 25 Glucose, 5 4-aminopyridine, pH 7.4 (adjusted with CsOH or MES), and the osmolarity was adjusted to 300 (±10) mOs-mol/L with glucose.

### Current-voltage Relationship

To record current-voltage relationship, after establishing whole-cell condition (with leak current <300 pA), cells were held at −100 mV and stepped to a range of potentials (−80 to +30 mV in 5 mV increments) for 50 ms. I-V curves were generated by plotting normalized peak currents (I/I_max_) as a function of depolarization potential. To obtain activation curves, peak currents (I) were first converted to conductance (G) at each voltage potential (V) by using the following equation: G = I/(V-V_rev_). V_rev_ is the reversal potential, which was determined for each cell individually. It is estimated by linear extrapolation of peak current amplitudes with depolarization potentials from 5 to 30 mV. Normalized conductance (G/G_max_) was plotted and fitted with Boltzmann’s equation: G/G_max_ = 1/{1+ exp[(V_1/2,act_-V)/k]}, where V_1/2, act_ is the voltage potential of half-maximal activation, and k is the slope factor.

### Steady-state Fast Inactivation

For steady-state fast inactivation study, cells were held at −100 mV and presented a 500 ms pre-pulse (ranging from −140 to +10 mV in 10 mV increment) followed by a 20 ms test pulse of −20 mV. The current measured from the test pulse was normalized with maximal current amplitude (I/I_max_) and plotted as a function of depolarizing potential. Plot was then fitted with Boltzmann’s equation: I/I_max_ = 1/{1+ exp[(V_1/2, inact_-V)/k]} to obtain the inactivation curve, where V_1/2, inact_ is the voltage potential of half-maximal inactivation, and k is the slope factor.

### Inactivation Recovery Rate

To analyze the rate of inactivation recovery, cells were held at −100 mV and a pair-pulse of −20 mV was applied. The time interval between the two pulses (interstimulus interval) was adjusted from 2 to 20 ms in 2 ms increment. The current recorded from the second pulse was normalized to the current recorded from the first pulse. Normalized current was plotted as a function of interstimulus interval and fitted with first-order exponential equation to obtain the recovery time constant, τ (tau).

### Drug Antagonism

For drug antagonism study, extracellular solutions with various concentrations of drugs were perfused through the recording chamber. Cells were held at −100 mV and stimulated with a 50 ms depolarizing pulse of −20 mV under treatment of drug and repeating the recording until the drug had been washed out. Percent of remaining current was then calculated as current recorded when treated with drug (I_drug_) divided by that after wash (I_wash_). The dose-response curve was fitted with the following logistic equation: I_drug_/I_wash_ = 1/[1+IC_50_/M]^n^, where IC_50_ is the concentration that results in 50% inhibition, M is the concentration of drug, n is the Hill coefficient. To assess use-dependent inhibition of mexiletine, cells were given repeated 50 ms depolarizing pulses of −20 mV from −100 mV holding potential at a frequency of 5 Hz in 6 seconds duration (total 30 pulses) with absence or presence of mexiletine. Current amplitude from each pulse was normalized to the maximal current (first pulse) measured with absence of mexiletine.

### Temperature Effect

The temperature of recording chamber was controlled at 25°C and 35°C with Badcontroller V (Luigs & Neumann, Ratingen, Germany). The accuracy of controller is ±0.1°C at 37°C, and ±0.2°C between 0°C to 60°C. The flow rate of external solution was 0.3 ml/min and the volume of recording chamber was approximately 3 ml. With such slow flow rate, the temperature fluctuation was minimal.

### Chemicals and Solutions

Lidocaine hydrochloride (L5647, Sigma-Aldrich Corp., MO, USA) and mexiletine hydrochloride (M2727, Sigma-Aldrich Corp., MO, USA) were dissolved in extracellular bath solution to give stock solutions of 100 mM. Subsequent dilutions were performed in extracellular bath solution to give concentrations of (mM): 0.1, 0.3, 1, 3, 10 and 30. Stock solutions were stored at 4°C for no more than three weeks. Working solutions were made fresh before patch-clamp recordings.

### Statistics

Statistics and curve fitting were done using Prism 5 (GraphPad Software, Inc., La Jolla, CA, USA), except for the IC_50_ curves, which were fitted using Origin 6.0 (OriginLab, Northampton, MA, USA). The unpaired t-test was used to compare data between groups and P<0.05 was considered statistically significant. Data are shown as means ± SEM.

## Results

### Genetic Analysis

Three missense mutations, one familial (I136V) and two sporadic mutations (I848T, V1316A) of *SCN9A* gene were identified in Taiwanese patients with primary erythromelalgia (PE). The familial mutation, isoleucine136 changed to valine (I136V), was previously reported [18], and its electrophysiological property was subsequently characterized by whole-cell patch clamp in HEK293 cells [19]. Isoleucine136 is located at the first transmembrane segment of domain I (DI/S1) of Na_v_1.7. In addition, two more cases, patient-A and –B were referred to our clinic without a positive family history. Patient A was a 33 year old female who developed excruciating burning pain at hot weather or in feverish at her young age. There were no symptoms of motor dysfunction. Although severe pain occurred, no hypo-, hyperesthesia or allodynia were found on her feet. She also denied any history of diabetes, autoimmune disease or Raynaud’s phenomenon. On examination, the feet were reddish and warm. There were no motor weakness, no muscle atrophy and no sensory loss on all sensory modality. The clinical electrophysiological data, including nerve conduction, variance of R-R interval and sympathetic skin response tests, were all unremarkable. The blood biochemistry and autoimmune profiles were normal. Given the typical phenotype of PE, sequencing of *SCN9A* gene in patient A showed a “T” to “C” substitution at nucleotide 2543 at exon 15 (reference sequence GeneBank NM_002977), which resulted in a missense mutation of isoleucine848 changing to threonine (Fig. 1A). The I848T is a de novo mutation since her parents do not harbor the sequence variant (Fig. 1A). Isoleucine848 is located in the intracellular linker region between the fourth and fifth transmembrane segments of domain II (DII/S4-S5) as shown in the upper panel of Fig. 1C. The same mutation was previously reported in three sporadic cases of Chinese patients and two familial cases (English and French) [6], [20], [21].

Patient B is also a sporadic case, who suffered from the typical phenotype of PE. The age of onset is at year 7 with progressive course implying a genetic disorder. She could not tolerate the severe feet burning pain but to immerse them with water or ice water. Inflammation with blisters and wounds on skin of both pedal regions ascending to mid-calf level further aggravated the erythromelalgia. Unfortunately, pain with autonomic instability resulted in neurogenic shock and she passed away after debridement. Sequencing of SCN9A gene in the patient identified a transversion of nucleotide “T” to “C” change at position 3947 (exon 22), but not in her parents (Fig. 1B). The novel mutation, V1316A, is located in the intracellular linker region between fourth and fifth transmembrane segments of domain III (DIII/S4-S5) (Fig. 1C).

Sequence alignments showed that the amino acids (I136, I848 and V1316), which mutated are highly conserved among human voltage-gated sodium channel α-subunit subtypes (Fig. 1A and 1B). They are also highly conserved among Na_v_1.7 homologs of various species during evolution. The conservation of amino acids at these positions suggest that mutations occur at these positions are likely to alter channel properties. These three mutations were not identified in 200 Taiwanese healthy controls.

### Current-voltage Relationship

To characterize the electrophysiological properties of these PE related mutant Na_v_1.7 channels, both wild type and mutant channels were over-expressed in CHO-K1 cells together with human SCN1B/2B β-subunits. The successful expression of Na_v_1.7 protein on the transfected cell membrane was demonstrated by Western blotting and immunofluorescent studies (Fig. S4 & S5).

**Figure 2:**
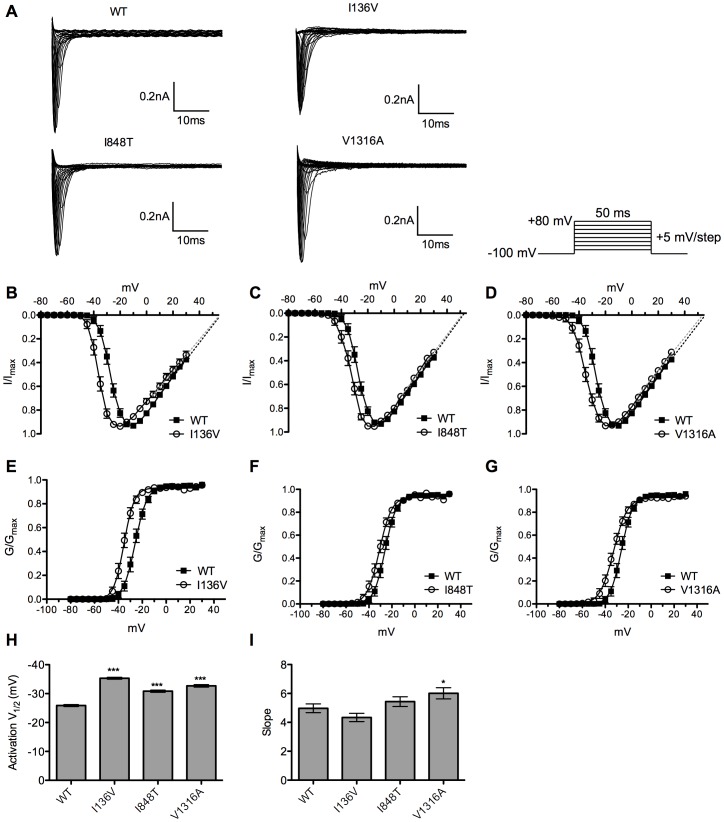
Current-voltage relationships of wild type and mutant Na_v_1.7 channels. (A) Representative current traces showed no apparent differences between wild type and mutant channels. (B–D) The current-voltage curves showed a hyperpolarizing shift in activation in all mutant (empty circles) channels without shifting in reversal potential as compared with wild type (solid squares). (E–G) The half maximal activation potential (activation V_1/2_) of each channel (mutant channel, empty circle and wild type channel, solid square). (H) The activation V_1/2_ of wild type channel is about −25.88 mV, but −35.32 mV for I136V, −30.84 mV for I848T, and −32.68 mV for V1316A mutant channel. (I) V1316A mutant channel shows an increase in slope factor and no difference in I136V and I848T mutant channels as compared with wild type. *P<0.05, ***P<0.001 vs. wild type; *t*-test; data shown as means ± SEM.

Using whole-cell patch clamp recording, the current-voltage relationships of wild type and mutant channels were recorded (Fig. 2). Cells were held at −100 mV and stepped to a range of potentials (−80 ∼ +30 mV with 5 mV increment) for 50 ms to record the current amplitude that was induced at each voltage step (Fig. 2A). The current-voltage (I-V) curves showed that there is a hyperpolarizing shift in voltage dependent activation for mutant channels with no apparent shift in reversal potential (Fig. 2B–D). Mutant channels were activated and reached maximal current amplitude at more hyperpolarized potential as compared with wild type channel. To obtain activation curves, normalized conductance was fitted with Boltzmann equation, and the half maximal activation potential (activation V_1/2_) of each channel was calculated (Fig. 2E–G). All mutant channels produce a significant (P<0.001) hyperpolarizing shift in activation V_1/2_ compared with wild type channel (−25.88±0.35 mV, n = 30) (Fig. 2H). I136V mutant channel has the largest shift (−35.32±0.33 mV, n = 26) among all mutant channels (I848T: −30.84±0.39 mV, n = 25; V1316A: −32.68±0.44 mV, n = 31). The slope factors of activation curves are similar except for V1316A mutant channel (6.01±0.39, n = 31), which has a significant (P<0.05) increase as compared with wild type (Fig. 2I). These results indicate an increase of channel sensitivity of voltage dependent activation in mutant channels.

### Steady-state Fast Inactivation Curves and Inactivation Recovery

**Figure 3:**
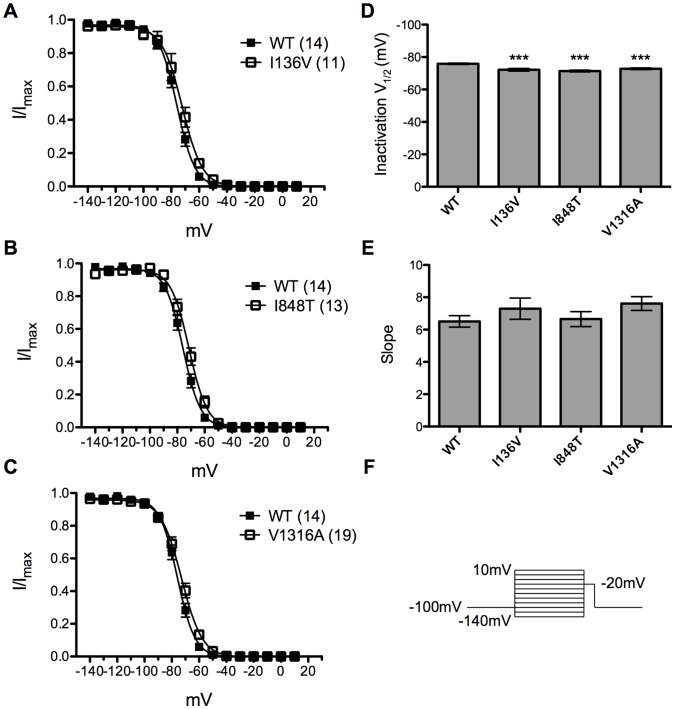
Steady-state fast inactivation curves of wild type and mutant Na_v_1.7 channels. (A–C) I136V, I848T, and V1316A mutant channels (open circles) produce a significant depolarizing shift in inactivation V_1/2_ as compared with wild type channel (solid squares). (D) Inactivation V_1/2_ of wild type channel is about −75.9 mV and mutant channels produce ∼3.7 mV depolarizing shift for I136V, ∼4.5 mV for I848T, and ∼3.1 mV for V1316A. (E) There is no significant difference in slopes. ***P<0.001 vs. wild type; *t*-test; data shown as means ± SEM.

Steady-state fast inactivation was tested by subjecting transfected cells to a series of 500 ms pre-pulses (−140 mV to −10 mV with 10 mV increment) and followed by a 20 ms test pulse of −20 mV (Fig. 3F). Boltzmann fits of normalized current amplitude from test pulses revealed significant (P<0.001) depolarizing shift in steady-state fast inactivation for all mutant channels compared with wild type (Fig. 3A–C). The half maximal inactivation potential (inactivation V_1/2_) of wild type channel was −75.89±0.41 mV (n = 14) (Fig. 3D). There was no apparent difference in slopes for all channels (Fig. 3E).

**Figure 4:**
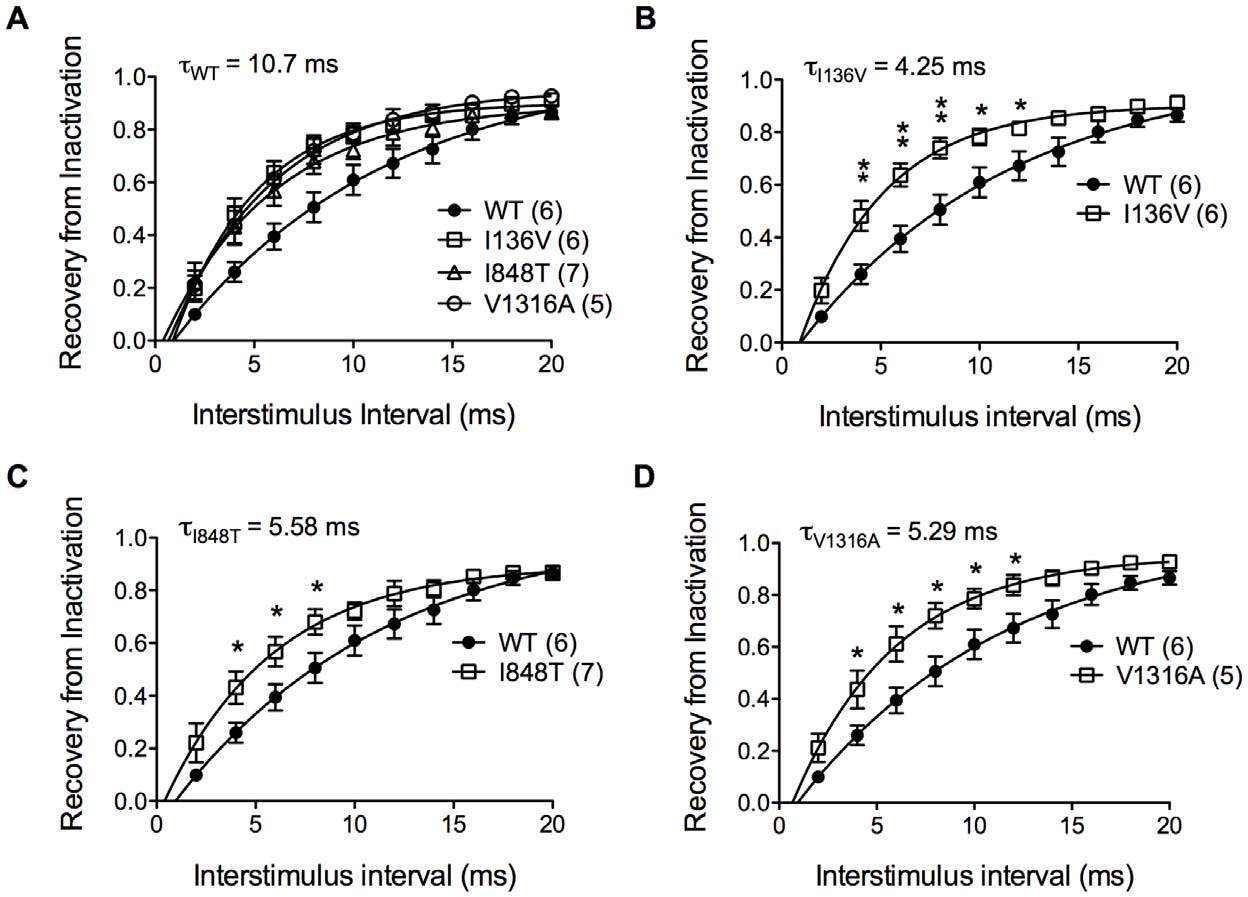
Inactivation recovery rate of wild type and mutant Na_v_1.7 channels. (A) Recovery time constant τ is 10.7 ms for wild type channel (solid circles). (B) I136V mutant channel has significantly faster recovery from inactivation between inerstimulus interval of 4–12 ms comapred with wild type channel and has a recovery τ of 4.25 ms. (C) I848T mutant channel recovers from inactivation significantly faster than wild type (solid circles) between 4–8 ms of interstimulus interval with a recovery τ, 5.58 ms. (D) V1316A mutant channel has significantly faster recovery from inactivation between interstimulus interval of 4–12 ms and has a recovery τ of 5.29 ms. N numbers are annotated in parentheses; *P<0.05, **P<0.01 vs. wild type; *t*-test; data shown as means ± SEM.

To assess the channel recovery rate from inactivation back to available state, a pair-pulse of depolarization from −100 to −20 mV was applied. The time interval between two pulses is the interstimulus interval and was adjusted from 2 to 20 ms with 2 ms increment. The percentage of current recovered from inactivation by the first pulse was measured and plotted as a function of time of interstimulus interval. The recovery time constant, τ (tau) was obtained by fitting the plot with first order exponential equation. The recovery τ was 10.7 ms (n = 6) for wild type channel (Fig. 4A). All mutant channels recovered significantly faster compared with wild type channel with recovery τ of 4.25 (n = 6), 5.58 (n = 7), 5.29 ms (n = 5) for I136V, I848T, and V1316A, respectively (Fig. 4B–D). The depolarizing shift of inactivation V_1/2_ and faster recovery would accentuate the channel conductance by allowing channel to remain open at more depolarized potential and being faster for ready to be reactivated.

### Temperature Effect

**Figure 5:**
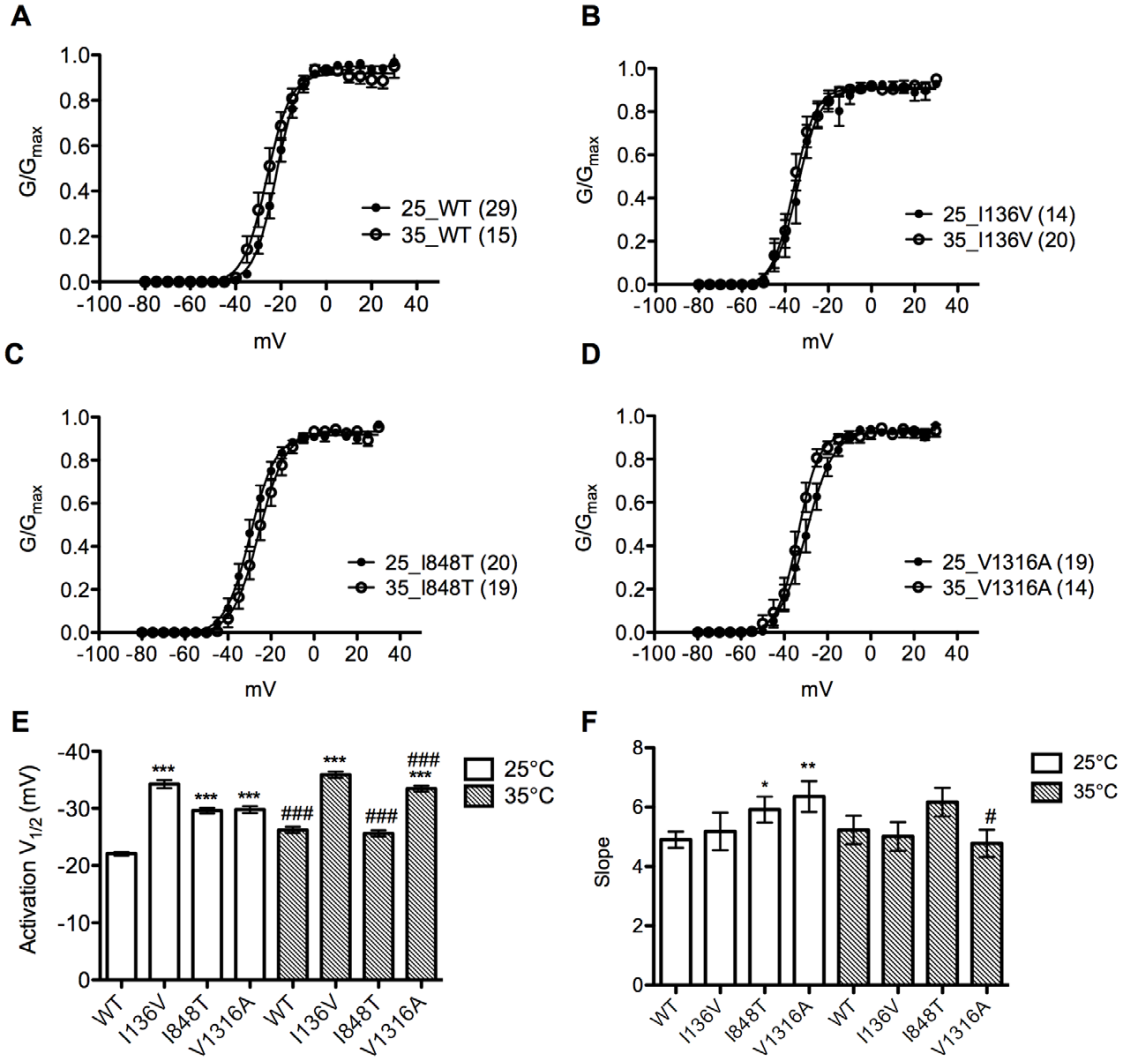
Activation curves of the mutant and wild type sodium channels at 25°C and 35°C. (A–D) Activation curves of wild type and I136V, I848T, V1316A mutant channels at 25°C (solid dots) and 35°C (open circles), respectively. (E) Wild type and V1316A mutant channels show a hyperpolarizing shift (WT: ∼4.2 mV; V1316A: ∼3.7 mV) in activation V_1/2_ at 35°C compared with 25°C, while I136V is unchanged. I848T mutant channel shows a depolarizing shift (∼4 mV) at 35°C. All activation V_1/2_ of mutant channels are more hyperpolarized than wild type at 25°C. (F) The slope of V1316A significantly reduces at 35°C as compared with 25°C. *P<0.05, **P<0.01, ***P<0.001 vs. wild type; ^#^ *P*<0.05, ^###^ *P*<0.001 vs. 35°C; *t*-test; data shown as means ± SEM.

Since warmth evokes and worsens symptoms and PE patients feel relieved after cooling, we therefore assessed the effect of temperature on wild type and mutant Na_v_1.7 channels. Both voltage dependent activation and steady-state fast inactivation were analyzed at 25°C and 35°C (Fig. 5, Table 1A & 1B). At 25°C, all the mutant channels had significant (P<0.001) hyperpolarizing shift (I136V: −34.24±0.72 mV, n = 13; I848T: −29.60±0.50 mV, n = 20; V1316A: −29.78±0.59 mV, n = 19) in activation V_1/2_ compared with wild type channel (−22.07±0.31 mV, n = 29) (Table 1A). Increase of temperature significantly (P<0.001) hyperpolarized the activation V_1/2_ of wild type (−26.22±0.55 mV, n = 15), and V1316A (−33.45±0.53 mV, n = 14) mutant channel (Table 1B). But, there was no effect on I136V mutant channel (Fig. 5B & 5D). However, both mutant channels still had more hyperpolarized activation V_1/2_ compared with wild type channel (Fig. 5E, Table 1B). On the contrary, I848T mutant channel produced a depolarizing shift of ∼4 mV (P<0.001, n = 19) and therefore resulted in no difference from wild type channel (Fig. 5C & 5E). The activation curves of I848T and V1316A at 25°C were less steep as compared with the wild type channel, whereas at 35°C there was no significant difference between mutant and wild type channels. V1316A mutant channel at 35°C had steeper slope as compared to that at 25°C (Fig. 5F).

**Table 1:** Summary of electrophysiological properties of wild type and mutant Na_v_1.7 channels at 25°C (Table 1A) and 35°C (Table 1B).

Table 1A						
Na_v_1.7	V_1/2, act_	Slope	n	V_1/2, inact_	Slope	n
WT	−22.07±0.31	4.90±0.27	29	−74.92±0.65	7.65±0.56	21
I136V	−34.24±0.72 ^***^	5.18±0.63	13	−71.84±0.99^*^	7.54±0.87	12
I848T	−29.60±0.50 ^***^	5.92±0.43^*^	20	−70.78±0.64^***^	8.38±0.56	21
V1316A	−29.78±0.59 ^***^	6.36±0.52^**^	19	−72.76±0.45^**^	7.22±39	20
**Table 1B**						
**Na_v_1.7**	**V_1/2, act_**	**Slope**	**n**	**V_1/2, inact_**	**Slope**	**n**
WT	−26.22±0.55^###^	5.23±0.48	15	−75.71±0.55	6.70±0.48	17
I136V	−35.89±0.56^***^	5.01±0.49	20	−68.4±0.82^***, #^	8.16±0.72	18
I848T	−25.62±0.55^###^	6.17±0.48	19	−67.52±0.54^***, ###^	7.20±0.47	21
V1316A	−33.45±0.53^***, ###^	4.78±0.46^#^	14	−70.88±0.61^***, #^	7.17±0.53	14

act = activation, inact = inactivation, *P<0.05, **P<0.01, ***P<0.001 as compared with WT; ^#^ *P*<0.05, ^##^ *P*<0.01, ^###^ *P*<0.001 as compared to the results in 25°C; *t*-test; data shown as means ± SEM.

**Figure 6:**
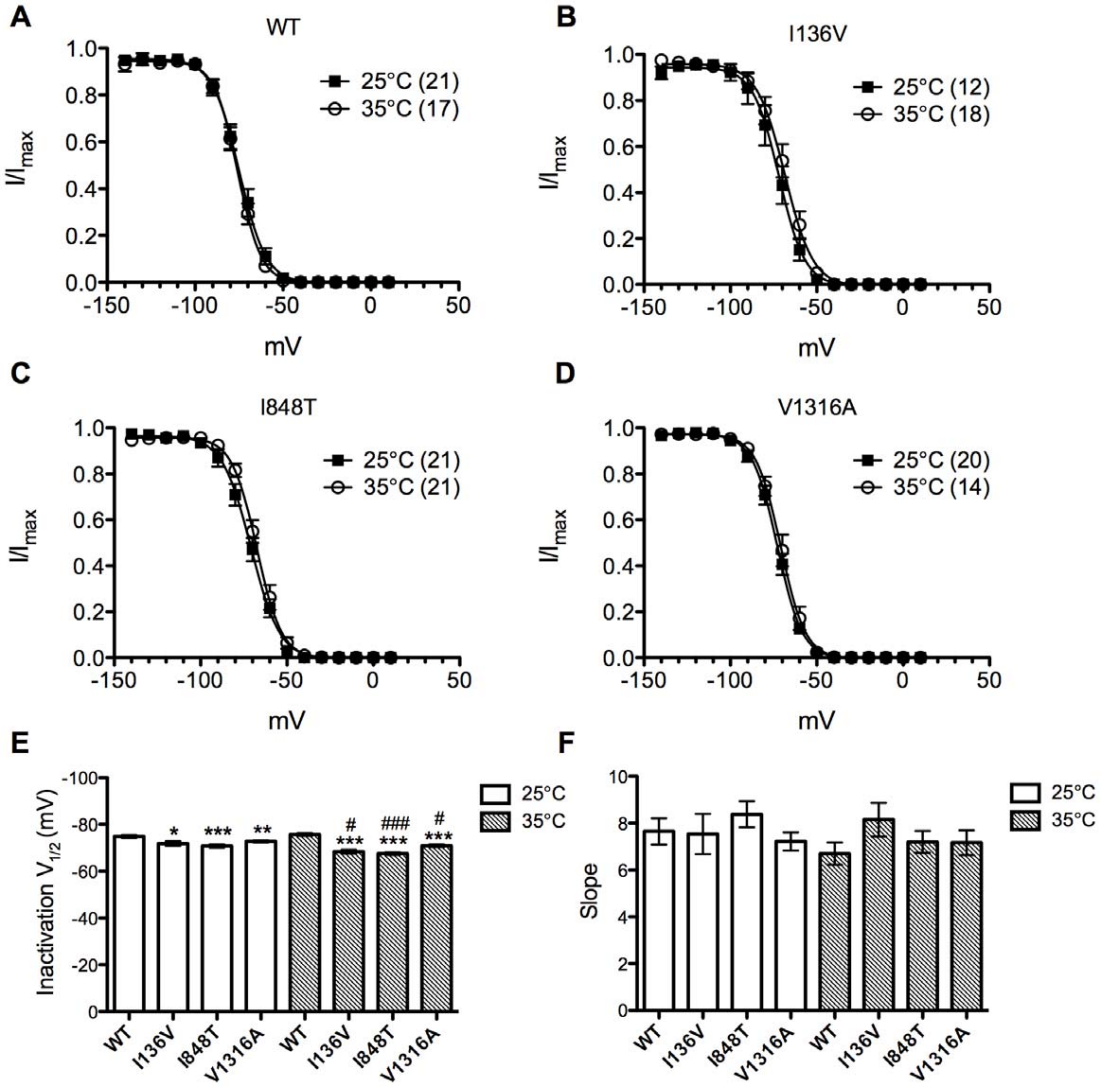
Steady-state fast inactivation curves of wild type and the mutant channels at 25°C and 35°C. (A–D) Steady-state fast inactivation curves of wild type and I136V, I848T, V1316A mutant channels at 25°C (solid squares) and 35°C (open circles), respectively. (E) No significant difference in inactivation V_1/2_ of wild type channel between 25°C and 35°C. At 35°C all mutant channels produce a significantly more depolarized inactivation V_1/2_ compared with 25°C. (F) There is no significant difference in slopes. *P<0.05, **P<0.01, ***P<0.001 vs. wild type; ^#^ *P*<0.05, ^###^ *P*<0.001 vs. 35°C; *t*-test; data shown as means ± SEM.

The steady-state inactivation in wild type channle was not affected by increase of temprature to 35°C (Fig. 6A). However, the inactivation V_1/2_ of all the mutant channels were further depolarized upon temperature increase (I136V: ∼3.44 mV, n = 18; I848T: ∼3.3 mV, n = 21; V1316A: ∼1.9 mV, n = 14) and therefore resulted in greater degree of shift compared with wild type channel (Fig. 6E). There was no significant difference in curve slopes. Thus, by increasing of temperature, channel conductance was enhanced especially in mutant Na_v_1.7 channels.

### Drug Antagonism

**Figure 7:**
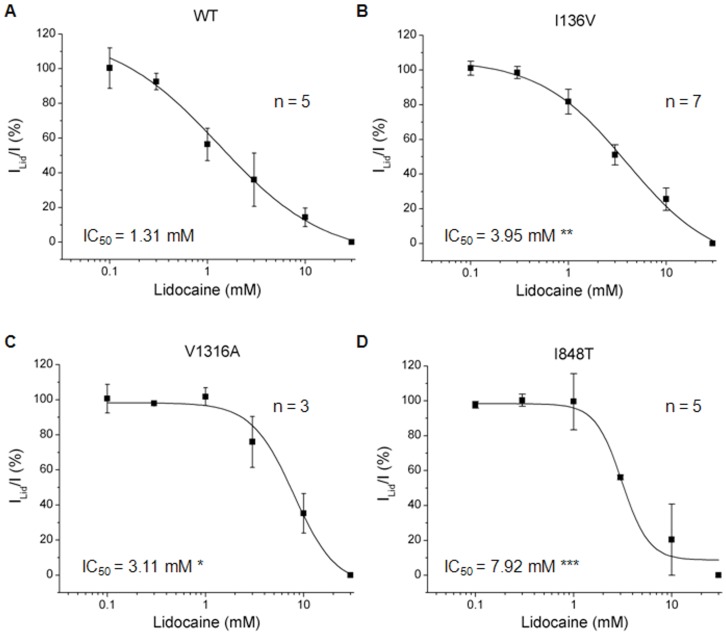
Lidocaine IC_50_ curves of wild type and mutant Na_v_1.7 channels. (A-D) Lidocaine IC_50_ curves of wild type, I136V, I848T, and V1316A mutant Na_v_1.7 channels, respectively. The IC_50_ value of wild type channel for lidocaine is 1.31±0.87 mM. All the mutant channels reveal significant higher IC_50_ values (I136V: 3.95±1.58 mM**; I848T: 3.11±0.48 mM*; V1316A: 7.92±2.50 mM***) compared with wild type. N for wild type is 5, I136V, 7, I848T, 3, V1316A, 4; *P<0.05, **P<0.01, ***P<0.001 vs. wild type; *t*-test; data shown as means ± SEM.

**Figure 8:**
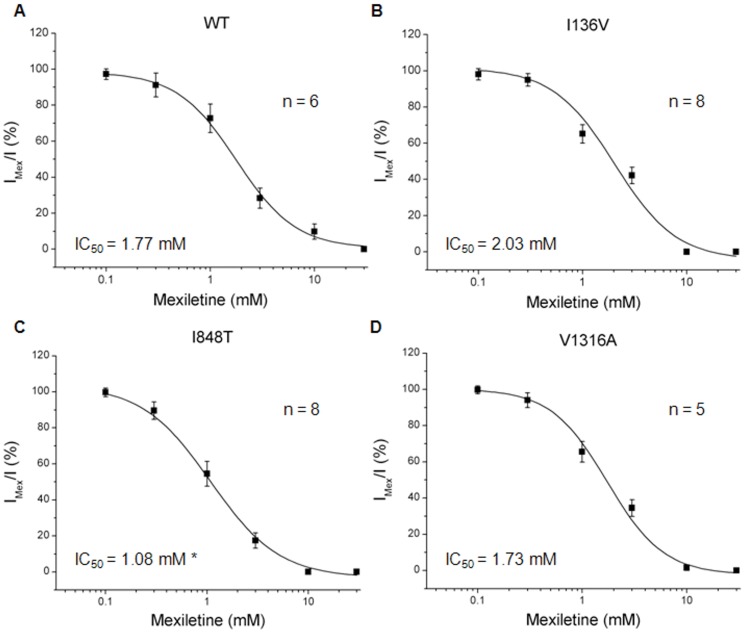
Mexiletine IC_50_ curves for wild type and mutant Na_v_1.7 channels. (A–D) Mexiletine IC_50_ curves of wild type, I136V, I848T, and V1316A mutant Na_v_1.7 channels, respectively. The IC_50_ value of wild type channel for mexiletine is 1.77±0.78 mM. No significant difference in IC_50_ values of I136V (2.03±0.38 mM) and V1316A (1.73±0.20 mM) mutant channels compared with wild type channel. I848T mutant has a significant (*P*<0.05) lower IC_50_ value (1.08±0.11 mM) compared with wild type channel. N for wild type is 6, I136V, 7, I848T, 7, V1316A, 8; *t*-test; data shown as means ± SEM.

Currently treatments for PE using sodium channel blockers such as local anesthetics (*e.g.* lidocaine), anti-convulsants (*e.g.* carbamazepine, phenytoin), or tricyclic antidepressants (*e.g.* imipramine) have only been partially successful. We sought to use electrophysiology to screen for drugs that are potentially effective for patients with these three mutations. Normalized peak currents induced by depolarizing from −100 to −20 mV with various concentrations of drugs were assessed and those showing 50% inhibition (IC_50_) were calcuated by fitting with logistic equation. We first tested the IC_50_ values of a local anesthetic, lidocaine, for wild type and mutant Na_v_1.7 channels. All three mutant channels seem to be more resistant to lidocaine blockade with significant higher IC_50_ values (I136V: 3.95±1.58 mM, n = 5; I848T: 3.11±0.48 mM, n = 7; V1316A: 7.92±2.50 mM, n = 4) compared with wild type channel (1.31±0.87 mM, n = 5) (Fig. 7). Furthermore, wild type channel had an IC_50_ value of 1.77±0.78 mM (n = 6) of mexiletine to inhibit half of the sodium conductance. The I136V (2.03±0.38 mM, n = 7) and V1316A (1.73±0.20 mM, n = 8) mutant channels revealed similar IC_50_ values as wild type channel (Fig. 8B & 8D), while I848T mutant channel resulted in a significant lower (1.08±0.11 mM, P<0.05, n = 7) IC_50_ for mexiletine (Fig. 8C). These results indicate that lidocaine is not be suitable for the patients; however, mexiletine might be an alternative choice, especially in the patient with I848T mutation.

## Discussion

In addition to reporting of a novel mutation V1316A, the electrophysiological properties for the three mutations (one familial, I136V and two sporadic, I848T & V1316A) were also evaluated in our study. Electrophysiologically, these mutations increased the sodium channel activity, which may result in hyperexcitability of sensory neurons. The hyper-activity of mutant Na_v_1.7 channels can be further enhanced at higher temperature. In search of effective treatment for PE, we found the differential inhibition of sodium channel blockers on these mutant Na_v_1.7 channels.

### Genetic Analysis

For electrophysiological study, we included all three mutations that were found in Taiwanese patients. Protein sequence alignments show that these amino acids (I136, I848, and V1316) are highly conserved during evolution and also among other voltage-gated sodium channel α-subunit subtypes, with an exception in I136 (Fig. 1A & 1B). I136V is located in D1S1 transmembrane segment (Fig. 1C), which has not been implicated in channel gating. However, it was shown that the S1– S3 segments form a narrow groove, which voltage sensor S4 slides along in an electric field by interacting with the negatively charged residues in neighboring transmembrane segments [22]. The substitution of isoleucine with valine might alter the conformation of the “groove” and as a result affect the voltage sensing and channel gating.

The missense mutation, V1316A is a novel mutation. The valine1316 is conserved among the species and sodium channel family (Fig. 1B). The I848T mutation has previously been identified in three sporadic case from China and two familial cases from England and France [6], [20]. It occurs most frequent as compared to all the other mutations in *SCN9A* gene resulting in PE (Fig. 1C). It occurs among variable ethnic groups of patients and could probably be a mutation hotspot. I848T and V1316A are both located on the intracellular loop linking transmembrane segment S4 and S5 of domain II and III respectively (Fig. 1C). Upon membrane depolarization, the voltage sensor S4 segment spirals outward and pulls on the S4/S5 linker, which induces movement of S5 and S6 segment and opens the channel pore [22]. Isoleucine848 is a nonpolar, neutral amino acid. Since threonine harbors a polar side chain, the replacing isoleucine by threonine might cause conformation change. Valine1316 is a nonpolar, neutral, and branched amino acid, while alanine is also nonpolar and neutral but structurally a much smaller amino acid. Both mutations, I848T and V1316A located on the S4/S5 linker might change the conformation of the linkers with S5 and S6 segments that is critical for channel gating. To authenticate a pathogenic mutation of these three sequence variants, the characterization of electrophysiological features would provide the direct evidence.

### Basic Electrophysiological Properties

The mutant Na_v_1.7 channels harboring the I136V, I848T, and V1316A mutations resulted in hyperpolarizing shift in voltage dependent activation and depolarizing shift of the inactivation V_1/2_ of steady-state fast inactivation (Fig. 2 & 3). Moreover, all three mutant channels recovered significantly faster from inactivation as compared with wild type channel (Fig. 4). Hyperpolarizing shift of activation threshold and the activation V_1/2_ would increase the channel sensitivity. Only V1316A mutant channel showed change in slope of the activation curve, which was less steep than wild type channel. This is due to the greater hyperpolarizing shift of activation threshold without much shift in the maximum current. Hyperpolarizing shift in activation together with the depolarizing shift in inactivation V_1/2_ might increase the window current which may result in depolarizing resting membrane potential of sensory neurons as previously demonstrated [23], [24]. The faster recovery from inactivation back to available state allows the mutant channels to be reactivated more rapidly than wild type channel, which contributes to hyper-activity of these mutant channels. Since no apparent difference was observed in reversal potential (Fig. 2B ∼ 2D), we reason that the changes in gating property is not due to permeation of other ions. Several mutations related to PE are located at the S4/S5 linker regions (Table 2), but there is no distinct property change in these regions compared with other mutations. It is still unclear why mutations located at different positions result in similar functional changes.

**Table 2:** Summary of Na_v_1.7 mutations located at S4/S5 linker regions.

Domain	Mutations	HEK293 Cell					DRG neuron	
		Activation V_1/2_	InactivationV_1/2, fast_	InactivationV_1/2, slow_	Deactivation	Ramp Current	AP threshold	Repetitive firing
I	I234T	Hyperpolarized	-	Hyperpolarized	Slowed	Increased	ND	ND
	S241T	Hyperpolarized	ND	Hyperpolarized	Slowed	Increased	ND	ND
II	I848T	Hyperpolarized	-	–	Slowed	Increased	Reduced	Enhanced
	L858H	Hyperpolarized	-	Hyperpolarized	Slowed	Increased	ND	ND
	L858F	Hyperpolarized	Depolarized	–	Slowed	Increased	Reduced	Enhanced
III	P1308L	Hyperpolarized	-	–	–	Increased	Reduced	Enhanced
	V1316A*	Hyperpolarized	Depolarized	ND	ND	ND	ND	ND

ND = Not determined, “−” = No difference compared with wild type, * = Novel mutation characterized in this study using CHO-K1 cells, AP = action potential.

Thus far, 17 mutations (as shown in Fig. 1C, except for N395K, Q875E, and novel mutation, V1316A) relating to PE have been characterized and all mutant Na_v_1.7 channels exhibit significant hyperpolarizing shift in voltage dependent activation compared with wild type channel [19], [23], [24], [25], [26], [27], [28], [29], [30], [31], [32], [33], [34], [35], [36], [37]. The mutant channels also produce depolarizing shift in steady-state fast inactivation [23], [24], [32], [34], [35], [37], slow inactivation [19], [25], [26], [28], [30], [31], [32], [33], [36], [37], slow deactivation [19], [24], [25], [26], [28], [29], [30], [31], [32], [34], [35], [36], and increased ramp current [19], [24], [25], [26], [27], [28], [29], [30], [31], [32], [34], [35], [36]. These channel property changes may confer to the hyperexcitibility in DRG neurons. Studies in DRG neurons showed that mutant Na_v_1.7 channels depolarized resting membrane potential [23], [24], lowered the threshold for action potential firing, and enhanced repetitive firing [23], [24], [26], [27], [33], [34], [37].

The electrophysiological data such as hyperpolarizing shift in voltage dependent activation for I136V and I848T mutant channels are comparable with previous studies, but not the steady-state fast inactivation [19], [30]. It has been reported that no significant shift in inactivation V_1/2_ in both I136V and I848T mutant channels except the steeper slope for I136V inactivation as compared with wild type channel. The discrepancy might be due to different expression systems. The previous studies were performed in HEK293 cells as oppose to CHO-K1 cells in our study. The HEK293 cells have a negative resting membrane potential and some cells express voltage-dependent sodium currents, whereas CHO-K1 cells we used have resting membrane potential close to 0 mV and do not express any detectable voltage-dependent sodium current. Additionally, the expression construct of Na_v_1.7 channel used by Cheng *et* *al*. (2008) and Cummins *et* *al*. (2004) was converted to TTX-R form, and electrophysiological recordings were conducted with TTX blockade of endogenous sodium current in HEK293 cells [19], [30]. The amino acid change for TTX sensitivity and different cell background might be the reason for different results. Therefore we only compared our findings to the wild type control in our system.

### Temperature Effect

It was reported by Babb et al. (1964) that the critical range of temperature, which triggers the painful attacks in erythromelalgia is 32–36°C [5]. Temperature is a critical factor to trigger the clinical features of PE. Han *et* *al*. found that cooling differentially shifts the midpoint of steady-state activation in depolarizing direction for L858F but not for wild type channels, which is likely to contribute to the alleviation of painful symptoms upon cooling in affected limbs in PE patients [34]. Therefore we assessed the gating property change in wild type and I136V, I848T, and V1316A mutant channels at 35°C compared to control of 25°C. In voltage dependent activation, our results showed that high temperature (35°C) could significantly hyperpolarize the activation V_1/2_ in wild type and V1316A mutant Na_v_1.7 channels but no change in the I136V one. The activation V_1/2_ of both I136V and V1316A mutant channels were still significantly more hyperpolarized as compared with wild type channel at 35°C. Surprisingly, I848T mutant channel shifted the activation V_1/2_ in depolarizing direction, which results in no significant difference from wild type channel at 35°C. The inactivation V_1/2_ of steady-state fast inactivation was not altered for wild type channel at 35°C. However, increasing temperature further depolarized the inactivation V_1/2_ of all three mutant channels.

Since wild type channel also exhibited hyperpolarizing shift in activation at 35°C, increasing temperature triggers the painful attacks in PE is probably due to the depolarizing of steady-state fast inactivation, which enhances the hyper-activity of mutant channels. Among three mutations, only V1316A mutant channel produced both hyperpolarizing shift in activation V_1/2_ and depolarizing shift in inactivation V_1/2_ while temperature shifts from 25°C to 35°C. These results are comparable to clinical observations while patient B (V1316A) presented with more severe symptoms as compared to the patients with the other two mutations. The depolarizing shift of voltage dependent activation observed in I848T mutant channel at 35°C is expected to counteract the depolarization in steady-state fast inactivation. However, the depolarization in activation does not result in further depolarizing shift as compared with wild type channel at 35°C and it is possible that the shift in steady-state fast inactivation alone contributes to the disease phenotype. The results could also be explained by the contribution of sympathetic nervous system, where Na_v_1.7 is also expressed abundantly [7].

Recent report has shown that neuropathic pain is resulted from the interaction between sympathetic and sensory neurons [38]. The effects of indifference and depolarizing shift of activation observed in I136V and I848T may be implicated in sympathetic neurons. Further characterizations in whether these mutations produce hyper- or hypo-excitability in sympathetic neurons, and how they interact with the hyperexcitable sensory neurons will help elucidating the pathophysiology of PE.

### Drug Antagonism

The differential inhibitions of clinically available medications for PE were tested on wild type and mutant Na_v_1.7 channels. The significant higher IC_50_ values for lidocaine observed in I136V, I848T, and V1316A mutant Na_v_1.7 channels suggest that lidocaine might not be a suitable treatment choice for patients with these mutations. In testing the mexiletine, only I848T mutant channel revealed a significant lower IC_50_ as compared with wild type channel. This result is compatible with the clinical observation that the visual analogue scale decreased in patient A (I848T) but not patient B (V1316A) after the mexiletine treatment. Although partial improvement was also observed in the patient carrying I136V mutation, there was no significant difference in IC_50_ as compared with wild type channel (Fig. 8B). Previous study on another PE relating mutation, V872G showed similar IC_50_ for mexiletine but exhibited greater use-dependent current fall-off as compared with wild type channel [29]. However, no difference was observed in our mutant channels compared with wild type channel in use-dependent assay (Fig. S6). Considering the disease phenotype might be resulted from interactions between sympathetic and sensory neurons [38], treatments that aim to attenuate the activity of Na_v_1.7 in sensory neurons would be expected to be only partially effective. Although our results do not completely match the clinical observation, our method to some degree can still provide a quick screen for potential treatments for these patients.

### Conclusions

Results from our study showed that the I136V, I848T, and V1316A mutant Na_v_1.7 channels exhibit altered electrophysiological properties that result in channel hyper-activity, which confers to the hyperexcitability in sensory neurons. Furthermore, we demonstrated that at higher temperature (35°C) to trigger PE symptoms, the hypersensitivity of mutant channels was accentuated and resulting in disease phenotype such as neuralgia and causalgia. Characterization of these mutant Na_v_1.7 channels in sensory and sympathetic neurons needs further investigation to unraveling the disease mechanism of PE.

## Supporting Information

Figure S1 **Cloning strategies of hSCN9A full-length cDNA.** (A) The positions of mutations are labeled in blue (I1376V), red (I848T), and green (V1316A) boxes along with the selected restriction enzyme sites. (B) Full-length hSCN9A was cloned into pTracer-EF/V5-His A vector using NotI cutting sites. (C) Fragments A/B/C containing mutation points were subcloned into pBluescript using indicated restriction enzymes (PstI/AvrII for fragment A; AvrII/AgeI for fragment B; AgeI/BsrGI for fragment C) for mutagenesis. Following successful mutagenesis, each mutation-containing fragment was cloned back to pTracer-hSCN9A.
(PDF)

Figure S2 **The restriction map for cloning of human** ***SCN1B*** **and** ***SCN2B*** **cDNA sequences.** The *SCN1B* was inserted flanking by the enzyme cutting site *MluI*. Under the same promoter for transcription, the *SCN2B* cDNA was cloned in the cutting site created by the enzymes, *SalI* and *NotI*.
(PDF)

Figure S3 **Fluorescent microscopy of the transfected CHO-K1 cells.** The successfully transfected cells demonstrated the bright green fluoscent protein, which were subjected for electrophysiological studies.
(PDF)

Figure S4 **Western Blotting for confirmation of the expression of SCN9A proteins in transfected cells.** To evalute the expression of SCN9A proteins, the transfected CHO-K1 cells were harvested for Western blotting. CHO-K1 cells transfected with *SCN9A* wild type and mutant constructs were lysed with 100 µl of 2X SDS sample buffer (125 mM Tris, 4% SDS, 20% Glycerol, 0.2% 2-ME, 0.001% bromophenol blue) in 35 mm dish. The lysate was centrifuged at 4°C for 10 min, and 15 µl of supernatant was subjected to SDS-PAGE. After being transferred to PVDF membranes, SCN9A wild type and mutant proteins were detected by anti-SCN9A antibody (Millipore, Billerica, MA, USA) with 1∶1000 dilution and visualized by enhanced chemiluminescence (ECL). The SCN9A protein expression were identified in the cells transfected with SCN9A constructs (among the wild type (WT) and the three mutants (I136V, I848T and V1316A), but not in the pTracer vector only (pTracer).
(PDF)

Figure S5 **Immunofluorescence imaging study demonstrated the membrane expression of the Na_v_1.7 channels in the transfected cells.** Cells were cultured on glass coverslip for 24 h after transfection, followed by fixation with 4% paraformaldehyde. The cells were not subjected to permeabilization procedure in order to observing the membrane expression. Cells were incubated with anti-SCN9A antibody (1∶100) (Millipore, Billerica, MA, USA) and detected by Cy3-conjugated secondary antibody (Millipore). Images were taken under a Carl Zeiss confocal microscope with appropriate excitation and emission filter pairs. On the upper panel, the expression of SCN9A were found on the membrane of CHO-K1 cells displaying red fluorescence. The membrane expression of Na_v_1.7 proteins was identified in the cells transfected with the wild-type (SCN9A WT) and the mutant clones (I136V, I848T and V1316A), but not with the vector only (pTracer).
(PDF)

Figure S6 **Use-dependent effect of mexiletine.** Wild type with both I136V and V1316A mutant Na_v_1.7 channels were treated with1 mM of mexiletine and present with high frequency stimuli (as decribed in methods). N numbers are annotated in parentheses.
(PDF)
