# Supplementary figures and images for: A Novel SCN9A Mutation Responsible for Primary Erythromelalgia and Is Resistant to the Treatment of Sodium Channel Blockers
Source: PLoS One. 2013 Jan 31;8(1):e55212. doi: 10.1371/journal.pone.0055212 (PMC3561374; doi:10.1371/journal.pone.0055212)

Supplementary

Figure S1A

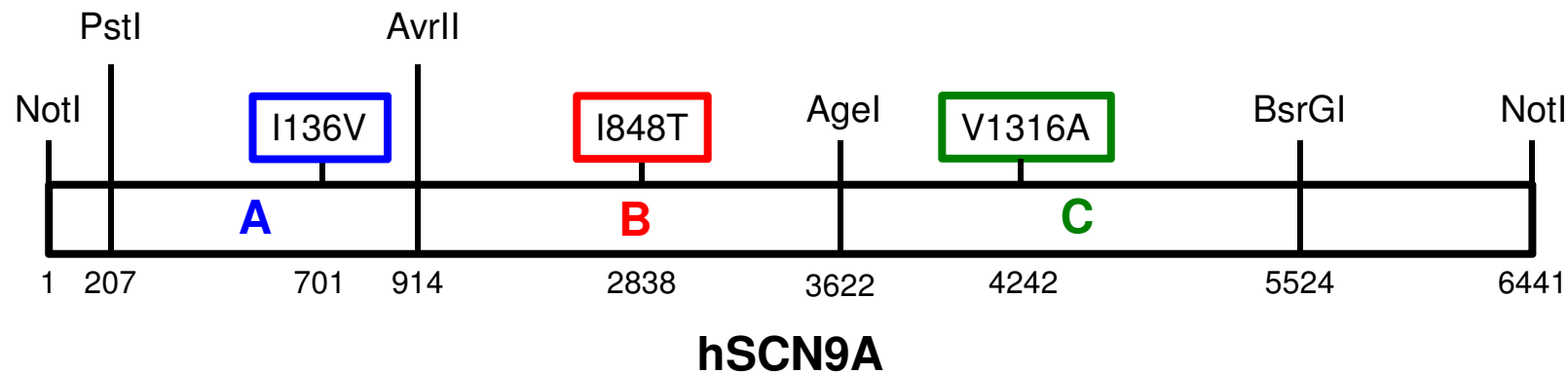

Figure S1B

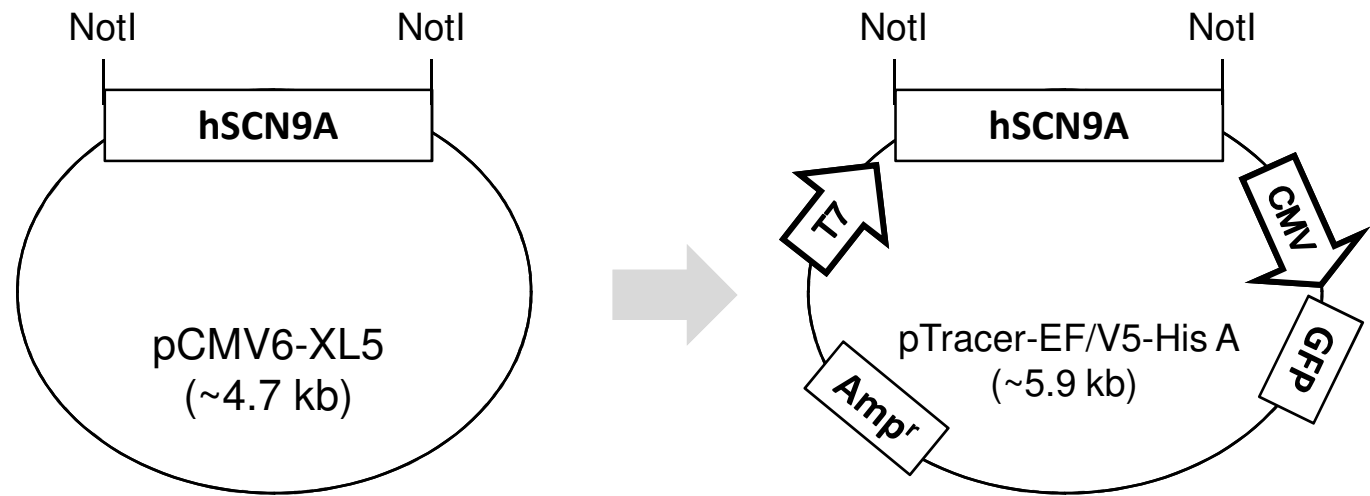

Figure S1C

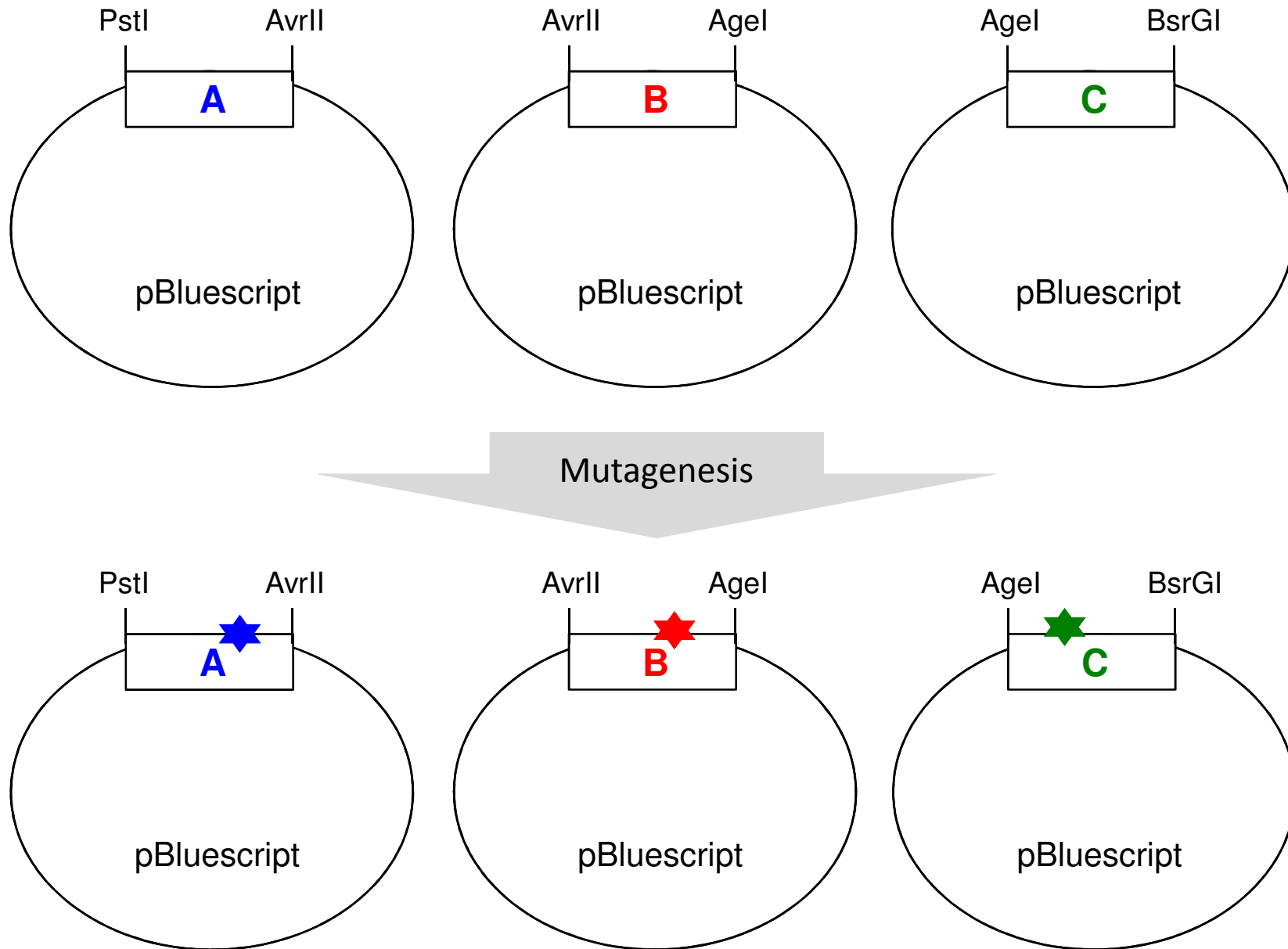

Supplement: Figure S1 — Cloning strategies of hSCN9A full-length cDNA. (A) The positions of mutations are labeled in blue (I1376V), red (I848T), and green (V1316A) boxes along with the selected restriction enzyme sites. (B) Full-length hSCN9A was cloned into pTracer-EF/V5-His A vector using NotI cutting sites. (C) Fragments A/B/C containing mutation points were subcloned into pBluescript using indicated restriction enzymes (PstI/AvrII for fragment A; AvrII/AgeI for fragment B; AgeI/BsrGI for fragment C) for mutagenesis. Following successful mutagenesis, each mutation-containing fragment was cloned back to pTracer-hSCN9A. (PDF) [file pone.0055212.s001.pdf]

Supplementary

Figure S2

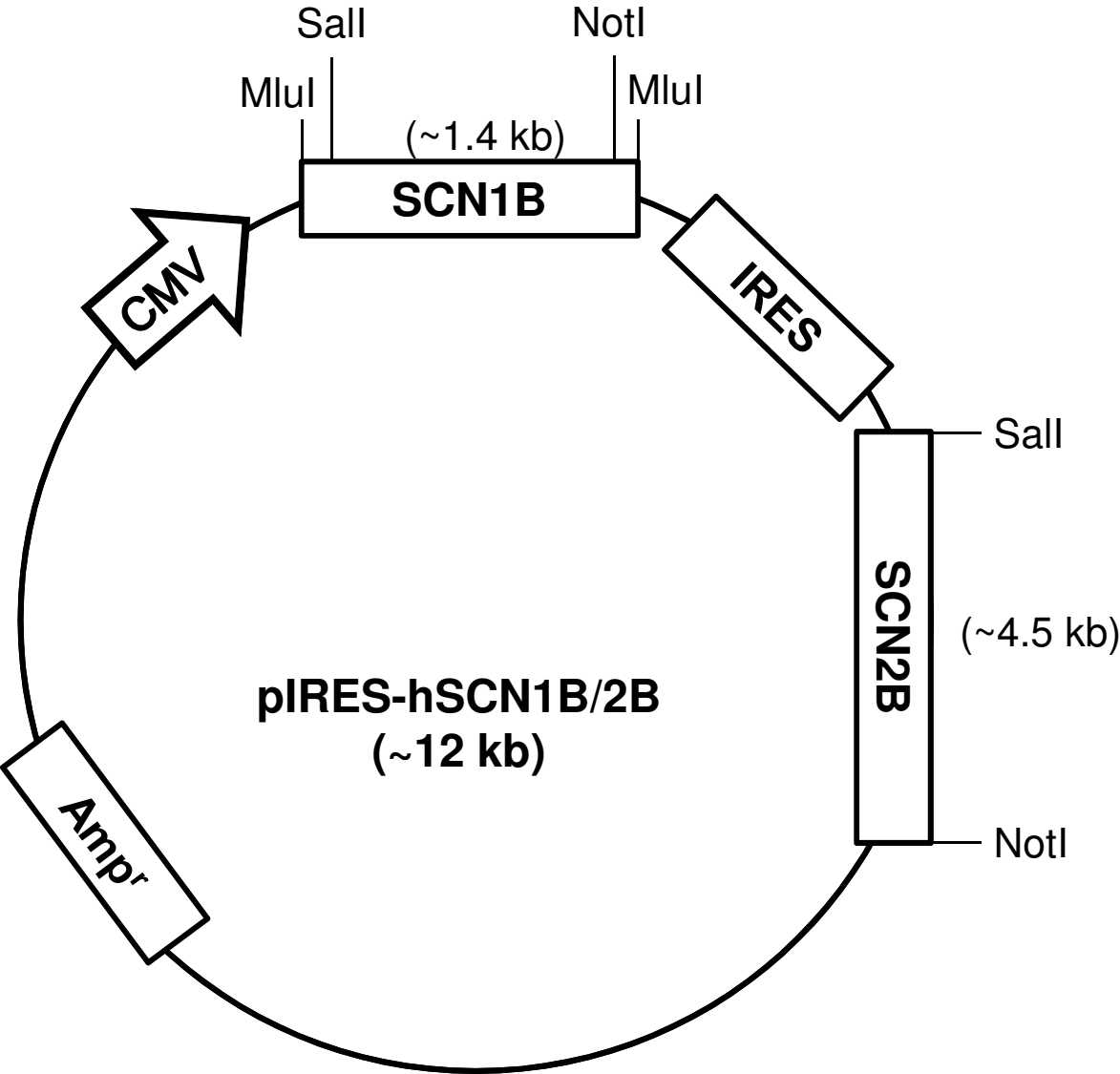

Supplement: Figure S2 — The restriction map for cloning of human SCN1B and SCN2B cDNA sequences. The SCN1B was inserted flanking by the enzyme cutting site MluI. Under the same promoter for transcription, the SCN2B cDNA was cloned in the cutting site created by the enzymes, SalI and NotI. (PDF) [file pone.0055212.s002.pdf]

Supplementary

Figure S3

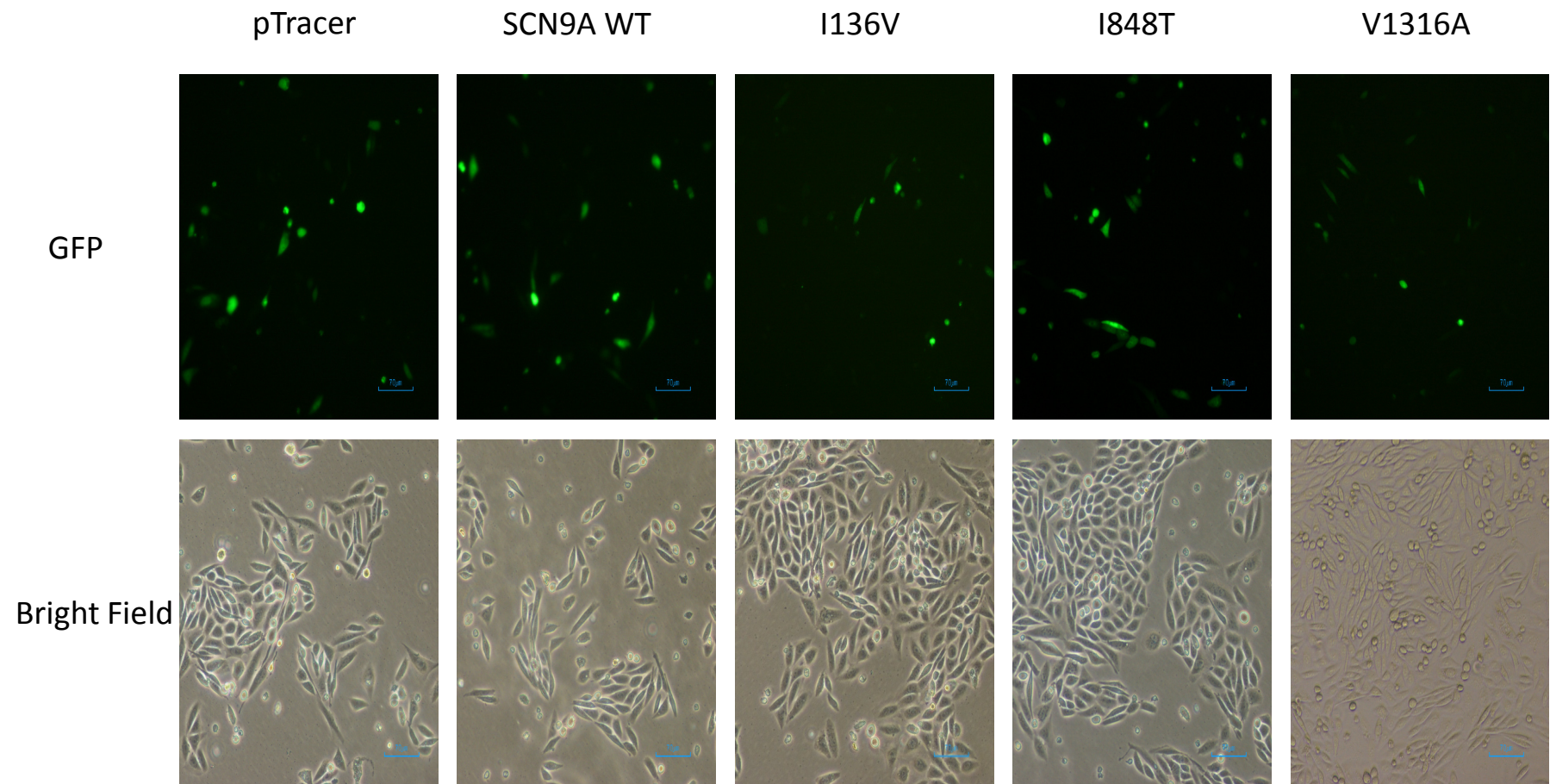

Supplement: Figure S3 — Fluorescent microscopy of the transfected CHO-K1 cells. The successfully transfected cells demonstrated the bright green fluoscent protein, which were subjected for electrophysiological studies. (PDF) [file pone.0055212.s003.pdf]

## Supplementary

Figure S4

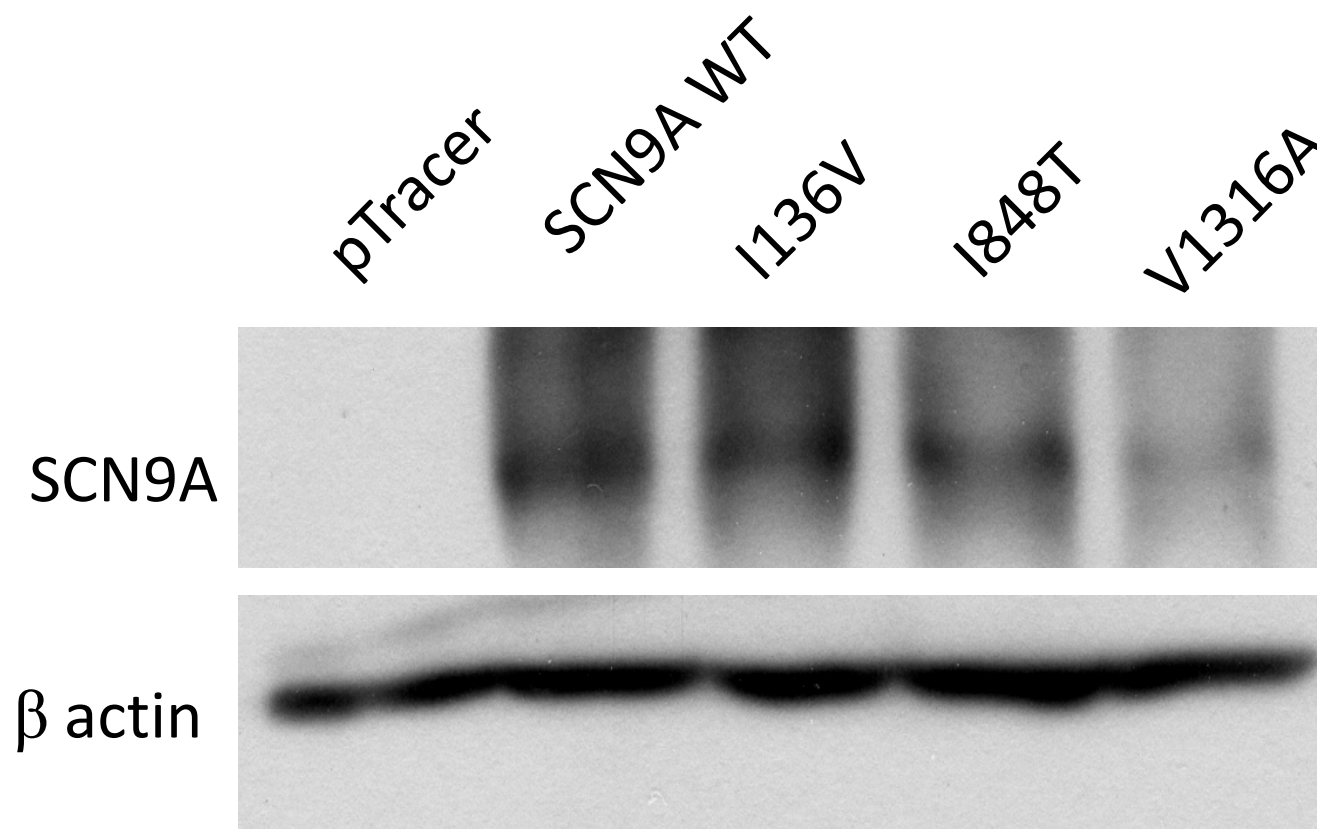

Supplement: Figure S4 — Western Blotting for confirmation of the expression of SCN9A proteins in transfected cells. To evalute the expression of SCN9A proteins, the transfected CHO-K1 cells were harvested for Western blotting. CHO-K1 cells transfected with SCN9A wild type and mutant constructs were lysed with 100 µl of 2X SDS sample buffer (125 mM Tris, 4% SDS, 20% Glycerol, 0.2% 2-ME, 0.001% bromophenol blue) in 35 mm dish. The lysate was centrifuged at 4°C for 10 min, and 15 µl of supernatant was subjected to SDS-PAGE. After being transferred to PVDF membranes, SCN9A wild type and mutant proteins were detected by anti-SCN9A antibody (Millipore, Billerica, MA, USA) with 1∶1000 dilution and visualized by enhanced chemiluminescence (ECL). The SCN9A protein expression were identified in the cells transfected with SCN9A constructs (among the wild type (WT) and the three mutants (I136V, I848T and V1316A), but not in the pTracer vector only (pTracer). (PDF) [file pone.0055212.s004.pdf]

## Supplementary

Figure S5

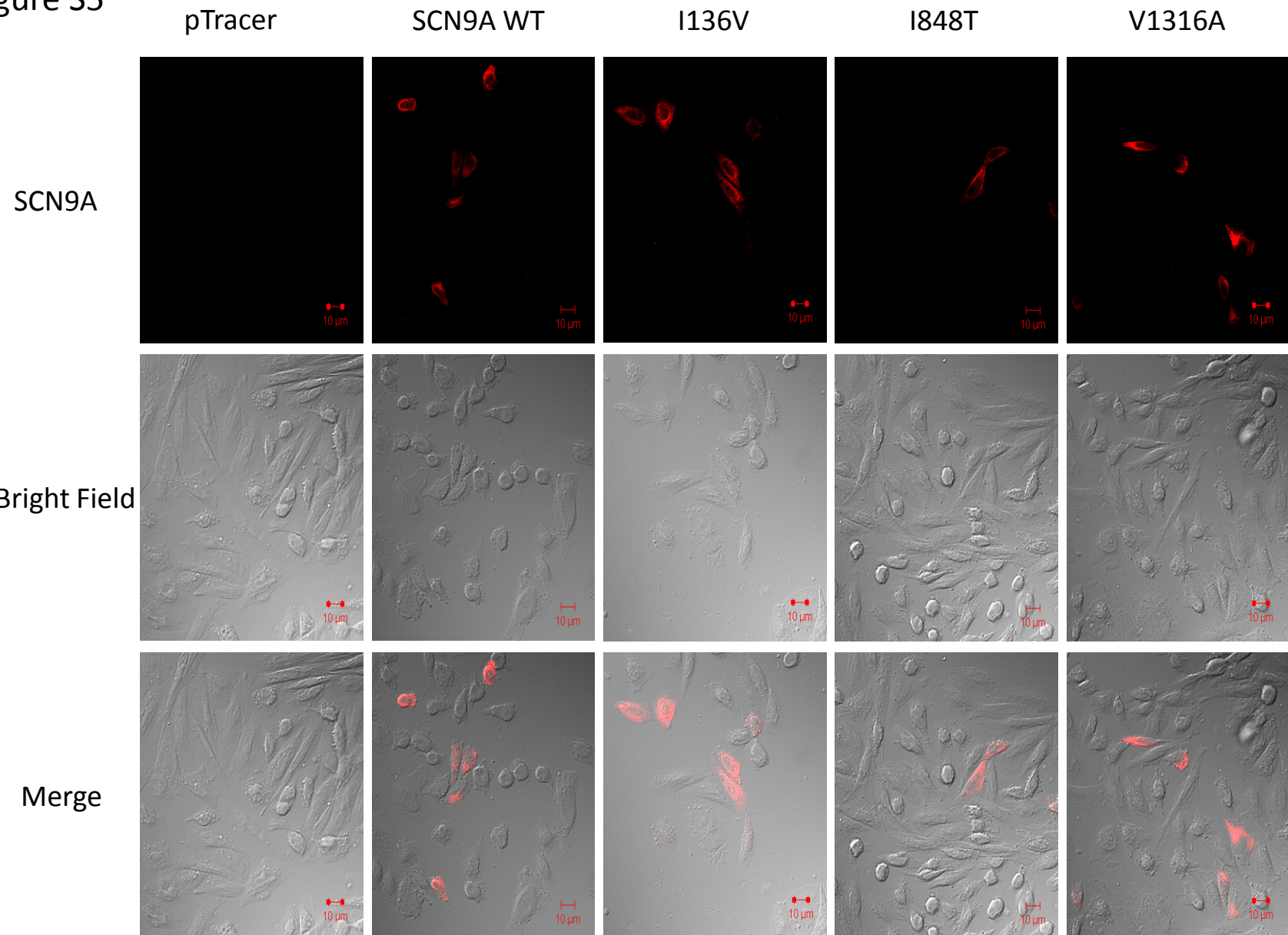

Supplement: Figure S5 — Immunofluorescence imaging study demonstrated the membrane expression of the Nav1.7 channels in the transfected cells. Cells were cultured on glass coverslip for 24 h after transfection, followed by fixation with 4% paraformaldehyde. The cells were not subjected to permeabilization procedure in order to observing the membrane expression. Cells were incubated with anti-SCN9A antibody (1∶100) (Millipore, Billerica, MA, USA) and detected by Cy3-conjugated secondary antibody (Millipore). Images were taken under a Carl Zeiss confocal microscope with appropriate excitation and emission filter pairs. On the upper panel, the expression of SCN9A were found on the membrane of CHO-K1 cells displaying red fluorescence. The membrane expression of Nav1.7 proteins was identified in the cells transfected with the wild-type (SCN9A WT) and the mutant clones (I136V, I848T and V1316A), but not with the vector only (pTracer). (PDF) [file pone.0055212.s005.pdf]

Supplementary

Figure S6

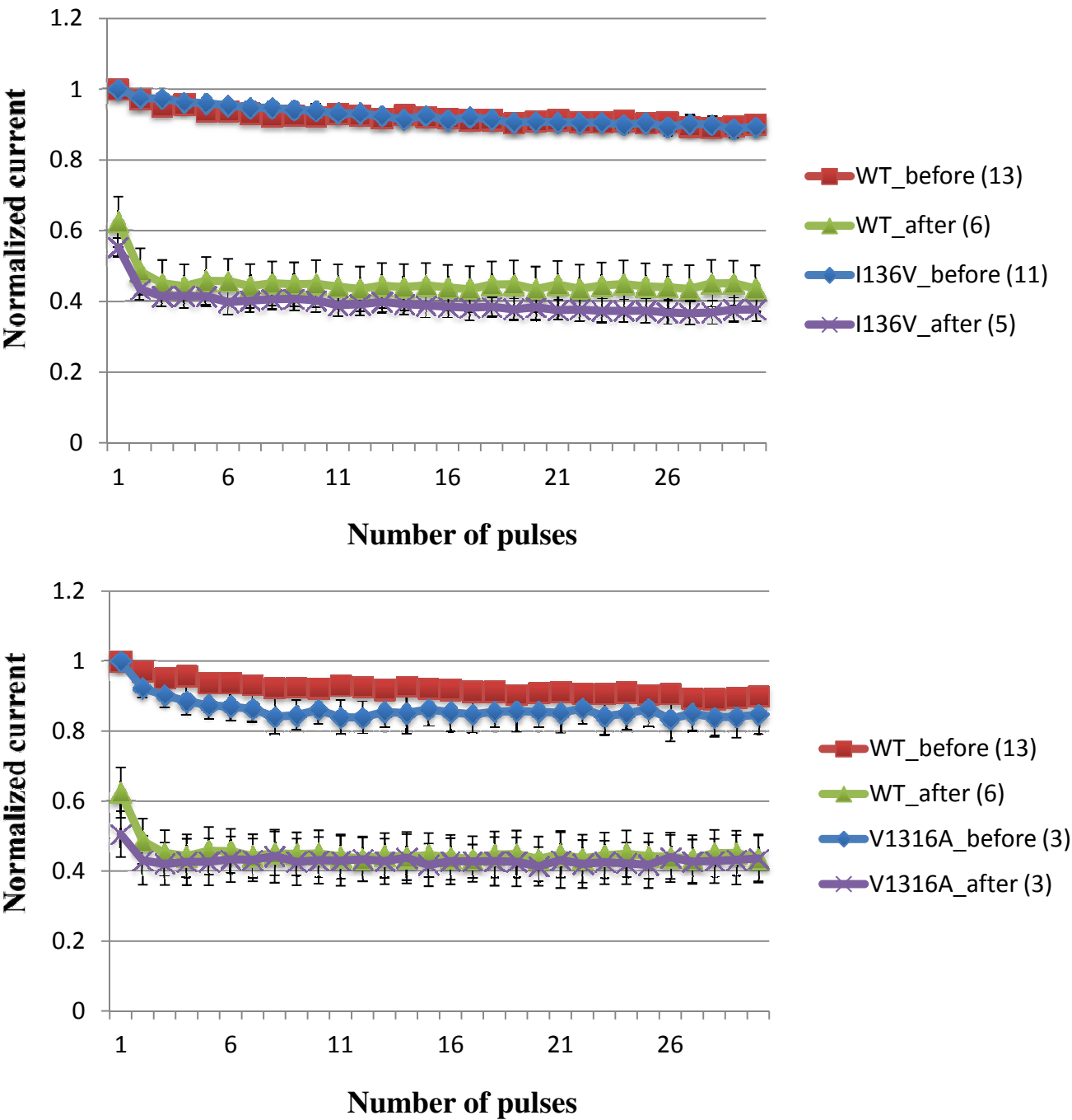

Supplement: Figure S6 — Use-dependent effect of mexiletine. Wild type with both I136V and V1316A mutant Nav1.7 channels were treated with1 mM of mexiletine and present with high frequency stimuli (as decribed in methods). N numbers are annotated in parentheses. (PDF) [file pone.0055212.s006.pdf]
